# Exposure and health risk assessment of nitrate contamination in groundwater in Coimbatore and Tirupur districts in Tamil Nadu, South India

**DOI:** 10.1007/s11356-020-11552-y

**Published:** 2020-11-10

**Authors:** Sajil Kumar Pazhuparambil Jayarajan, Lemoon Kuriachan

**Affiliations:** 1 Institute of Geological Sciences, Hydrogeology Group, Freie Universität Berlin, Malteserstr. 74-100, 12249 Berlin, Germany; 2 Water Institute, Karunya University, Coimbatore, 641114 India

**Keywords:** Groundwater quality, Nitrate contamination, Human health risk assessment (HHRA), Crystalline aquifer, South India

## Abstract

Anthropogenic factors are contaminating crystalline aquifers more rapidly than natural sources and affecting human health in many states in India. Since a large population depends on untreated groundwater, identifying the sources of this contamination and assessing the related human health risk are essential to ensure a good-quality water supply. Nitrate is one of the most widespread means of groundwater contamination in many parts of India. Coimbatore and Tirupur districts are the most rapidly growing industrial urban areas in southern India. This paper deals with nitrate contamination and possible health risks for children and adults in the Coimbatore and Tirupur districts based on 93 groundwater samples. To achieve this goal, classical hydrochemical and deterministic hazard identification methods coupled with spatial mapping technologies were applied. A wide variation in nitrate concentration, between 1 and 415 mg/L, was observed, with 37% of the samples exceeding the WHO permissible limit of 50 mg/L. The distinct concentrations of nitrate and other ions observed spatially can be attributed to the diverse geochemical and land use settings in the study area. The bivariate plots of NO_3_ with other ions suggested that the principal origin of nitrate in this study is related to the excess application of fertilizers and sewages. The spatial variation of NO_3_, in comparison with the land use map, confirmed these results. The values of hazard quotient (HQ) via ingestion exceeded the critical value, one in 40% in males, 42% in females, and 45% in children. However, HQ values via oral pathways are within one and pose no exposure risk. Thus, the hazard index corresponds to HQ_ingestion_ only. The health risk was in the increasing order of male>female>children, and shows that body weight is the most critical factor that is influencing the health impact to children as compared to adults. The spatial variation of hazard index values showed that groundwater quality is highly polluted with NO_3_ in the north and northeastern parts of the study area, mainly due to intensive agricultural practices, and poses critical health concerns. Considering the increasing population and higher dependencies on groundwater, immediate and sufficient measures are proposed.

## Introduction

Groundwater is a vital source of water which supports human health, agriculture, and industrial development and plays a critical role in the sustainability and functioning of ecosystems due to a lack of clean surface water (Ali and Ali 2018; Steube et al. 2009). The industrial revolution and the rise in anthropogenic activities over the past decades have increased the need for groundwater more than ever before (Kaviarasan et al. 2016). The uncontrolled growth in population provokes a rapid increase in socioeconomic activities, which may emit contaminants into the environment, thus posing a threat to groundwater quality. Fertilizers, effluent runoff from industries, chemical dumping, and domestic sewage are considered to be the primary sources of groundwater pollutants and waterborne diseases (Nalbantcilar and Pinarkara 2016). Approximately 25,000 people around the globe die every day due to lack of water or consumption of polluted water, and nearly one-third of the urban population in developing countries lacks safe drinking water (Li and Ling 2006). Of all the groundwater pollutants, NO^−^_3_ (nitrate) has emerged as one of the most dangerous and widespread contaminants of groundwater in arid and semi-arid regions (Adimalla 2019; Chen et al. 2016; Chica-Olmo et al. 2016; Li et al. 2019).

Nitrate is a form of nitrogen and originates naturally in the environment from atmospheric nitrogen, final ion by plants, and lightning storms. It can also appear as the result of anthropogenic activities like fertilizers, septic tanks, sewage, and improper use of animal manures for agriculture. NO_3_ is innocuous to human beings, and its formation is a fundamental part of the nitrogen cycle (Shukla and Saxena 2018). Nitrate, which is ingested through drinking water, however, is reduced from nitrate to nitrite in the gut by bacteria. This causes methemoglobinemia, or more generally known as a blue baby syndrome in new-born babies, a life-threatening condition that decreases the ability of blood to carry oxygen through the body. Nitrite, converted from nitrate, can also react with organic compounds to form amines and amides through intrastation in the stomach, and the interaction of nitrous acid with secondary and tertiary amides, amines, and nitrogen-containing compounds forms N-nitroso compounds, widely considered to cause gastric cancer. In addition, there are many other diseases such as thyroid dysfunctions, breathing problems, nuisance and tiredness, and multiple sclerosis that can result from high nitrate intake (Ahada and Suthar 2018; Gatseva and Argirova 2008; Vanhatalo et al. 2018; WHO 2011).

Groundwater NO^−^_3_ contamination is highly discussed around the globe and has become a predominant threat because of the uncontrolled use of pesticides (Shukla and Saxena 2018; Spalding and Exner 1993). In India, nitrate contamination is an emerging issue regarding groundwater safety and human health. It is reported that one-third of the states in India have excess nitrate in their drinking water, and Tamil Nadu is one of them (NEERI 1991; Taneja et al. 2017). Nitrate fertilizer usage in India has tremendously increased over a period of 55 years from 1951 to 2006 (Adimalla 2019). Because of a low level of retentiveness of NO^−^_3_ by the soil and a high level of water solubility, the excess amount of NO^−^_3_, unutilized by the plant, gets easily leached to the sub-soil layer. This leached NO_3_ is finally reaching groundwater table and traveling in the same velocity of groundwater (Ahada and Suthar 2018). Nitrate concentration is very high in arid and semi-arid areas, though much lower in deep groundwater as compared to shallow groundwater (He et al. 2020).

Many studies have been done on the risk to human health associated with nitrate-contaminated groundwater in India. Adimalla (2019) studied the exposure risk and health impacts of nitrate in Nirmala province, South India, and reported that the health of the women and children are profoundly affected. Ahada and Suthar (2018) carried out a study in the southern district of Punjab to assess NO_3_ contamination and showed that 92% of the area has a higher level of NO_3_ than the safe limit. Another study in the Shanmunganadhi river basin also reported that children are more vulnerable than adults for health risks from elevated levels of nitrate (Karunanidhi et al. 2019).

According to the WHO, the maximum allowable limit of nitrate in drinking water is 50 mg/L (WHO 2017). A higher concentration of nitrate than this level has already been reported in some parts of the area covered in this study (Sajil Kumar et al. 2014). However, no study covering the entire Coimbatore and Tirupur district has been published to date. The need for this study is essential because there is a growing need for greater agricultural activities to feed an increasing population. The population growth is promoting urbanization and exponentially increasing settlements in the study area. All these factors affect nitrate levels in groundwater and deteriorate the groundwater quality. In this background, a detailed study was conducted to understand the track of nitrate and to determine the human health risk of nitrate exposure for children and adults (men and women). Furthermore, to quantify the quality of the groundwater, a drinking water quality index (DWQI) was developed. Thus, this data can be used as an assessment guideline for the decision-makers in groundwater quality protection and management.

## Study area settings

**Fig. 1:**
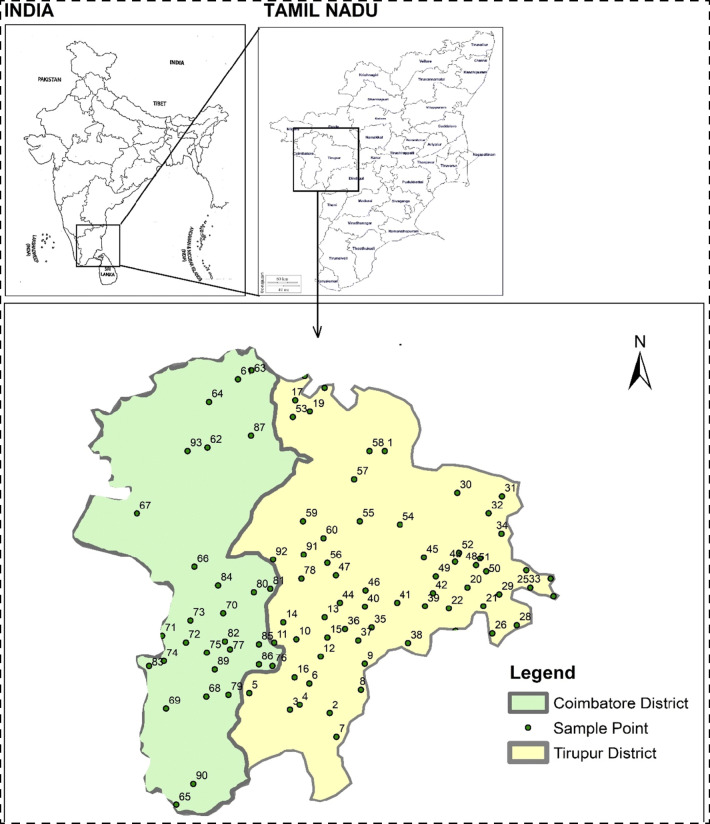
Location map of the study area showing the Coimbatore and Tirupur districts and sample points

The location map of the study area and the sampling wells are shown in Fig. 1. Coimbatore district is bordered by the Western Ghats in the west and Tirupur district in the eastern boundary. According to the CGWB (2008) report, the climate of this region is sub-tropical, with a range of temperatures from 14 to 40 °C. The primary rainy season is the SW and NE monsoon seasons which together contribute around 650 mm of rainfall per year. Bhavani, Noyyil, and Amaravathy are the main rivers, but are usually dry in the summer season. The topography is mostly undulating, and the slope of the terrain is mainly to the east. The lithology of the Coimbatore district is comprised of metamorphic rocks, charnockites, granites, hornblende–biotite gneiss, sillimanite gneiss with basic and ultra-basic intrusive, crystalline limestone, syenite, pegmatite, and quartz veins. Sedimentary formations such as alluvium and kankar deposits are found in the Noyyal and Bhavani river basins. Of the rock types, hornblende–biotite gneiss is the most abundant. The geomorphology of the district includes structural and residual hills, linear ridges, Bazada zones, buried piedmonts, active pediments, shallow piedmonts, erosional plains, and valley fills (CGWB 2008). Red calcareous soil, black soil, red non-calcareous soil, alluvial and colluvial soil, brown soil, and forest soil are the common soil types. Most of the aquifers are crystalline rocks from the Archean age and with water stored in the fissures, fractures, joints, and weathered portions. Along the Noyyal river course, the sedimentary formations also act as water-bearing formations. Weathered hard rocks are up to 30 m thick and are the prominent water storage medium in the study area.

Tirupur is the nearby district of Coimbatore district which is added at a later stage to the state of Tamil Nadu. The climate is also tropical in nature with a temperature of 20 to 40 °C. The district receives little rainfall as it is situated along the leeward side of the Western Ghats (Arumugam and Elangovan 2009). The NE monsoon is the most important source of groundwater recharge as the SW rains are negligible. Lithology follows the same pattern as in Coimbatore. Dug wells and bore wells are the essential methods of water extraction. In the past few decades, the subsidized power supply for the farming villages has affected the groundwater table through the overexploitation of groundwater resources.

## Materials and methods

### Groundwater sampling and analytical techniques

A total of 93 groundwater samples were collected in July of 2016 from the Coimbatore and Tirupur districts in Tamil Nadu and were analyzed at the water quality laboratory of Institute of Water Studies (IWS), Chennai. These wells were selected after a reconnaissance survey which considered the importance of representing the exact hydrogeological condition of the aquifers. Water was pumped out before sampling to stabilize the physicochemical parameters. Samples were collected in pre-cleaned polyethylene bottles, sealed, and brought to the laboratory for analysis. Samples were kept at 4 °C until the analysis was completed. Standard analytical procedures, as suggested by APHA (1995) for the analysis of groundwater samples, were followed throughout. Only highly pure (AnalR Grade) chemicals and double-distilled water were used for preparing solutions for analysis. Electrical conductivity (EC) and pH were analyzed using portable digital meters in the field. Calcium (Ca^2+^) and magnesium (Mg^2+^) were determined using EDTA titration. Chloride (Cl−) was determined by standard AgNO_3_ titration. Carbonate (CO_3_^2−^) and bicarbonate (HCO_3_^−^) were determined by titration with HCl. Sodium (Na^+^) and potassium (K^+^) were measured by flame photometry. Sulfate (SO_4_^2−^) was determined by using a UV–visible spectrophotometer. Nitrate was measured by ion chromatography. The values were observed to be within the standard limit of ± 5%.

### Drinking water quality index

Based on the physicochemical analysis of pH, total dissolved solids (TDS), NO_3_, Ca, Mg, Na, K, Cl, SO_4_, CO_3_, HCO_3_, F, and TH, the DWQI was calculated. These parameters were assigned weights (wi) between 1 and 5, according to their importance and health effects (Sajil Kumar et al. 2019). The highest weight of five has been given to F and NO_3_¯, as they are highly toxic even with very low concentration. A weight of four has been assigned to pH, Cl, Na, and Ca, and the other parameters were assigned a medium weight of three or less. Based on the assigned weights, the relative weights for each parameter are calculated using Eq. 1, and the values are shown in Table 1.

1 $$ \mathrm{Rw}i=\frac{\mathrm{wi}}{\sum \limits_1^n\mathrm{wi}} $$

**Table 1:** Weights assigned and the calculated relative weights for groundwater quality parameters

Parameter	Weight (wi)	Rwi
pH	3	0.08
TDS	4	0.11
TH	2	0.05
Ca	3	0.08
Mg	2	0.05
Na	3	0.08
K	2	0.05
Cl	3	0.08
SO4	4	0.11
HCO3	2	0.05
NO3	5	0.13
F	5	0.13
Total	38	1

In this equation,

RWi: is the relative weight
Wi: is the rate of each parameter
*n*: is the number of parameters

In the next step, the quality rating scale (qi) has been calculated using Eq. 2

2 $$ \mathrm{qi}=\left(\mathrm{Ci}/\mathrm{Si}\right)\times 100 $$

In this equation,

qi: is the quality rating
Ci: is the concentration of ions in mg/L
Si: is the drinking water quality standard (WHO 2017)

Then, the sub-index (SI) was calculated using Eq. 3, allowing the DWQI to be calculated with Eq. 4.

3 $$ \mathrm{SI}i=\mathrm{Rwi}\times \mathrm{qi} $$

4 $$ \mathrm{DWQI}=\sum \mathrm{SI}i $$

### Human health risk assessment

The toxic elements affect human health, mainly by inhalation, ingestion, and dermal pathways. A high concentration of nitrate in water can cause serious health issues. In this study, the health impact of nitrate on males, females, and children has been investigated. The standard procedures and reference values suggested by the United State Environmental Protection Agency (USEPA 2001) are used in the health risk assessment. Exposure risks by ingestion and dermal pathways were calculated using Eq. 5 and Eq. 6:

5 $$ {\mathrm{CDD}}_{\mathrm{IN}}=\left({C}_{\mathrm{water}}\times \mathrm{IR}\times \mathrm{EF}\times \mathrm{ED}\right)/\left(\mathrm{BW}\times \mathrm{AT}\right) $$

6 $$ {\mathrm{CDD}}_{\mathrm{DE}}=\left({C}_{\mathrm{water}}\times \mathrm{SA}\times \mathrm{KP}\times \mathrm{EF}\times \mathrm{ED}\times \mathrm{ET}\times \mathrm{CF}\right)/\left(\mathrm{BW}\times \mathrm{AT}\right) $$

Here, CDD_IN_ and CDD_DE_ represent the chronic daily dose by ingestion and dermal effects, respectively (μg/kg day). *C*_water_ is the concentration of NO_3_ in groundwater (mg/L). IR is the ingestion rate of water in L/day (adults = 2.5 L/day; children = 0.78 L/day). SA is the exposed skin area in cm^2^ (adults = 16,600 cm^2^; children = 12,000 cm^2^). KP is the dermal permeability coefficient for water (0.001, no unit). EF is the water exposure frequency (365 days). ED is the exposure duration in years (males = 64, females = 67, and children = 12,). ET is the water exposure time in hours/day (0.4 h/day for adults and children). BW is the body weight in kg (men = 65, women = 55, and children = 15). AT is the average residence time measured in days/year. CF is the conversion factor (0.001 for adults and children).

The hazard quotient (HQ) of NO_3_ exposure via ingestion and dermal pathways can be calculated using Eq. 7 and Eq. 8 below:

7 $$ {\mathrm{HQ}}_{\mathrm{IN}}={\mathrm{CDD}}_{\mathrm{IN}}/\mathrm{RfD} $$

8 $$ {\mathrm{HQ}}_{\mathrm{DE}}={\mathrm{CDD}}_{\mathrm{DE}}/\mathrm{RfD} $$

HQ_IN_ is the ingestion-based hazard quotient. HQ_DE_ is the dermal-based hazard quotient. RfD is the reference dose of NO_3_, i.e., 1.6 mg/kg/day (USEPA 2014).

Hazard index (HI) is the overall risk of exposure via both digestion and dermal pathways.

9 $$ \mathrm{HI}={\mathrm{HQ}}_{\mathrm{ingestion}}+{\mathrm{HQ}}_{\mathrm{dermal}} $$

If the HI values are higher than one, it may cause non-carcinogenic health hazards.

### Spatial mapping using inverse distance weighting

The distribution of ions in different locations can be easily visualized and interpreted by plotting them on spatial variation maps. The spatial analyst tool in ArcGIS has several interpolation techniques. One of the easiest and useful methods is inverse distance weighting (IDW), used in this study. The basic assumption is that when several known points are distributed through space, the points near them are mostly similar than those falling away. The main advantage is that the method is logical and efficient. Since our sample points are mostly evenly distributed, this study has adopted the IDW method. The weights for unknown points may be determined using the following equation,

$$ {\lambda}_i=\frac{D_i^{-\alpha }}{\sum_{i=1}^n{D}_i^{-\alpha }} $$

where *λi* is the weight of point, *D*_*i*_ is the distance between point “*i*” and the unknown point, and *α* is the power ten of weight.

## Results and discussion

### Groundwater quality

The statistical summary of the major physio-chemical parameters and their permissible limit for drinking purpose is shown in Table 2. One of the significant operational water quality parameters is pH, which requires an optimum range of 6.5 to 8.5 (WHO 2017). In the samples from this study, pH varied between 7 and 9, with a mean value of 8. This indicates that the groundwater of the study region is slightly alkaline. The pH value is controlled by carbonates and bicarbonates in the aquifer (Saha and Paul 2019). EC indicates the total amount of dissolved salts in the water; in physical terms, it is the capacity of water to convey electric current. In the present study, EC ranged from 180 to 7290 μS/cm, with an average value of 1811 μS/cm. TDS measure the total concentration of dissolved substances in the water. TDS is a combination of inorganic salts and a small amount of organic matter (Li et al. 2008; Sajil Kumar 2014). TDS values range from a minimum value of 100 to a maximum value of 4439 mg/L. The average value of TDS in the present study is 1103 mg/L. According to the classification of TDS suggested by Todd (1980), samples 91, 62, and 4 fall under the category of very freshwater (0–250 mg/L); 60% of samples fall under the category of freshwater (250–1000 mg/L); and 37% of sample fall under the category of brackish (1000–10,000 mg/L), with none of the samples in the category of saline (10,000 to 100,000 mg/L).

**Table 2:** Statistical summary of water quality parameters of 92 wells in the study area

Parameter	Minimum	Maximum	Average
pH	7.4	8.9	8.26
EC	180	7290	1811.18
TDS	100	4439	1103.15
TH	60	2000	526.77
Ca	10	700	87.462
Mg	6.075	291.6	74.87
Na	7	690	145.89
K	4	360	43.23
Cl	14	2092	275.18
SO_4_	2	816	115.92
CO_3_	0	60	11.68
HCO_3_	69.29859	603.9	276.59
NO_3_	1	415	76.92
F	0.2	2.16	1.163

In major anions, the order of abundance was found to be Cl˃HCO_3_^−^˃SO_4_^−^˃NO_3_^−^˃F^−^. Chloride in the groundwater may generally come from anthropogenic sources such as runoff containing road de-icing salts, inorganic fertilizer usage, effluents from septic tanks and industrial activities, animal feeds, and seawater intrusion in coastal areas (Han et al. 2014). The value of chloride in the present study was observed to be between 14 and 2092 mg/L with a mean value of 275 mg/L, which is above the acceptable limit of 250 mg/L (WHO 2017). A high concentration of chloride gives drinking water a salty taste. Bicarbonate concentration varied between 69 and 604 mg/L, with an average value of 277 mg/L. The concentration of bicarbonate remained mostly within the limit of 500 mg/L. The sulfate concentration varied between 2 and 816 mg/L, with an average of 116 mg/L. Samples 1, 11, 35, 40, 41, 44, 47, 53, 75, and 81 were found to exceed the allowable limit of 250 mg/L (WHO 2011). The range of fluoride in groundwater was between 0 and 2.0 mg/L, with an average of 1 mg/L. Together, 19% of the total samples exceed the permissible limit of 1.5 mg/L (WHO 2011).

The dominance of the major cations was in the order of Na^+^˃Ca^+^˃Mg^2+^ ˃K. Na is the dominant ion among the cations, and its concentration ranged between 7 and 690 mg/L, with a mean value of 146 mg/L. The permissible limit of Na^+^ in drinking water is 200 mg/L. In total, 24% of the samples showed that the level of Na^+^ exceeded this permissible limit. Sample 75 showed the highest value, measuring 690 mg/L. Ca^+^ and Mg^2+^ directly contribute to water hardness and are the most abundant elements in groundwater. The Ca and Mg concentrations in the water samples from the present study varied from 10 to 700 mg/L, avg. 87 mg/L, and 6 to 292 mg/L, avg. 75 mg/L, respectively. The Ca^2+^concentration of all samples, except samples 11, 13, and 81, was within the permissible limit of 300 mg/L. The permissible limit for Mg^2+^ of 150 mg/L was exceeded by 15% of the total samples. K^+^ varied between 4 and 360 mg/L, with an average of 43 mg/L, remarkably higher than the permissible limit of 12 mg/L.

Total hardness is defined as the sum of calcium and magnesium ions in water. In the present study, the lowest value of 60 mg/L was found in sample 61 and the highest value of 2000 mg/L was found in sample 11. The average value of the total hardness was 527 mg/L, which is higher than the desirable limit of 300 mg/L. According to Sawyer (1967), 30% of the samples fall under the category of hard (150–300 mg/L) and 70% under the category of very hard (˃ 300 mg/L), while none was found to fall under the category of soft (0–75 mg/L) or moderately hard (75–150 mg/L).

### Drinking water quality index

DWQI or drinking water quality index is a mathematical operative tool which presents a single number for the overall quality of drinking water (Mohebbi et al. 2013). This can help in the decision-making process of administrative representatives for better planning and management of groundwater resources (Sajil Kumar et al. 2019). According to the DQWI method, the physicochemical analysis data of the present study was classified into excellent water (˂ 50), good water (50–100), poor water (100–200), very poor water (200–300), and water unsuitable for drinking when the DWQI exceeds the value of 300 (Mohebbi et al. 2013). A detailed classification of the DWQI for this study is given in Table 3.

**Table 3:** Results and classification of groundwater quality based on WQI

Water quality	Range	Number of samples	Percentage
Excellent water	˂ 50	22	23%
Good water	50–100	35	37%
Poor water	100–200	25	26%
Very poor water	200–300	10	10%
Unsuitable for drinking	˃ 300	3	3%

In the present study, the computed DWQI value ranges between 26 and 361. The average value of the DWQI is 114, which is classified as poor water quality for drinking purposes. In total, 23% of the samples have DWQI values below 50, indicating excellent quality, and 36% of the samples ranged between 51 and 99, categorized as good water. On the other hand, 26% of the samples showed very poor quality for drinking purposes. Sample 1 at Timmanaickanpalayam village gave the highest value of 361, followed by sample 11 at Pudupalayam village with the value 319. In sample 1, the DWQI value was high due to the high level of TDS (2173 mg/L), and other ions such as NO_3_ (366 mg/L), Cl (425 mg/L), SO_4_ (260 mg/L), and K (360 mg/L). The presence of a high concentration of NO_3_ and K shows that the pollutants originate from agricultural fertilizers.

**Fig. 2:**
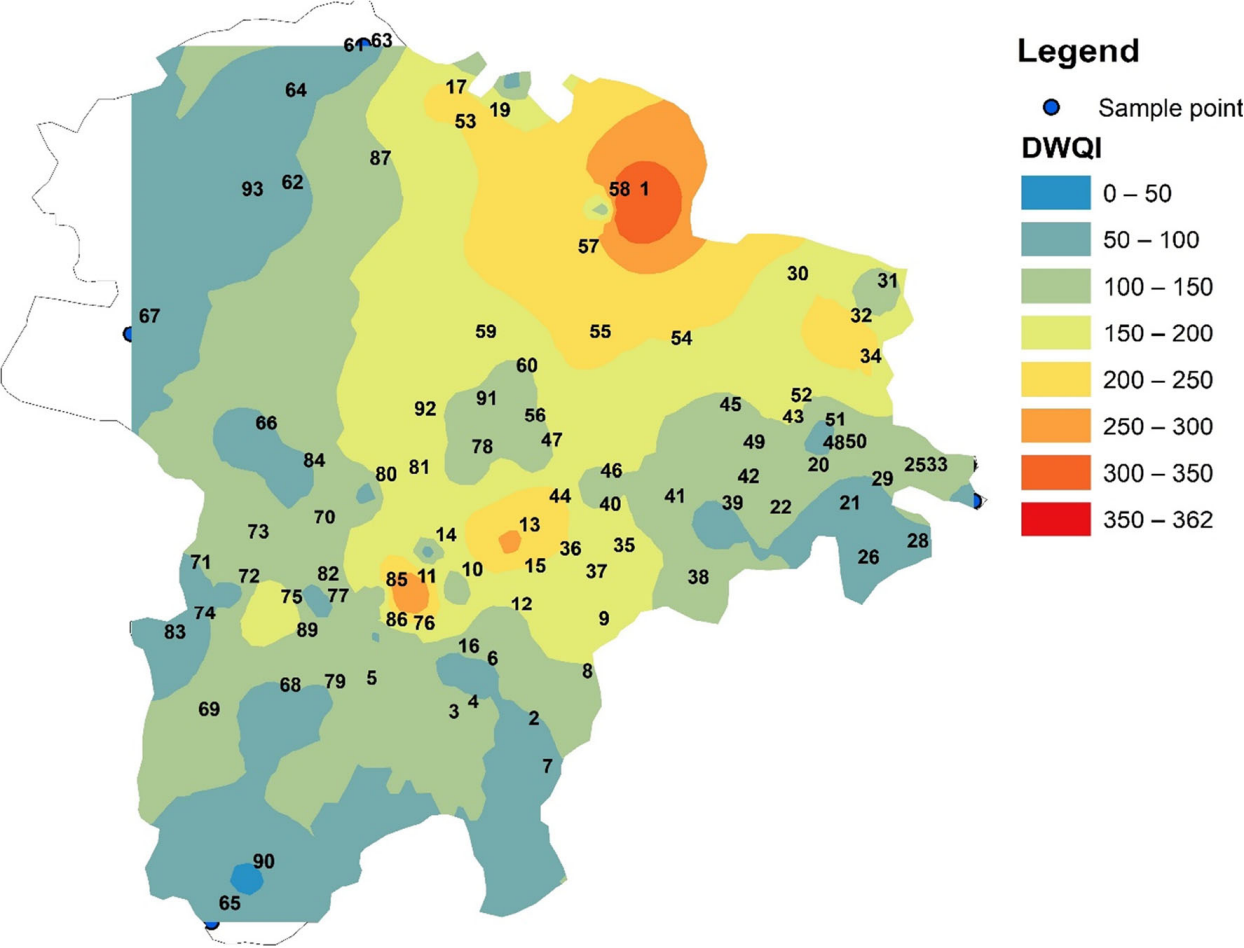
Spatial variation of the drinking water quality Index (DWQI) in the study area

The spatial variation map of the DWQI showed that the samples in the southern, eastern, and northeastern regions have comparably better-quality water than the northern and northwestern regions (Fig. 2). Some samples in the central part and majority of the samples in the northwestern region have very high DWQI values, categorized as poor or very poor. The samples in these regions are mostly affected by anthropogenic inputs such as industrial or agricultural activities. The land use pattern of the study area agrees with this inference.

### Groundwater types

**Fig. 3:**
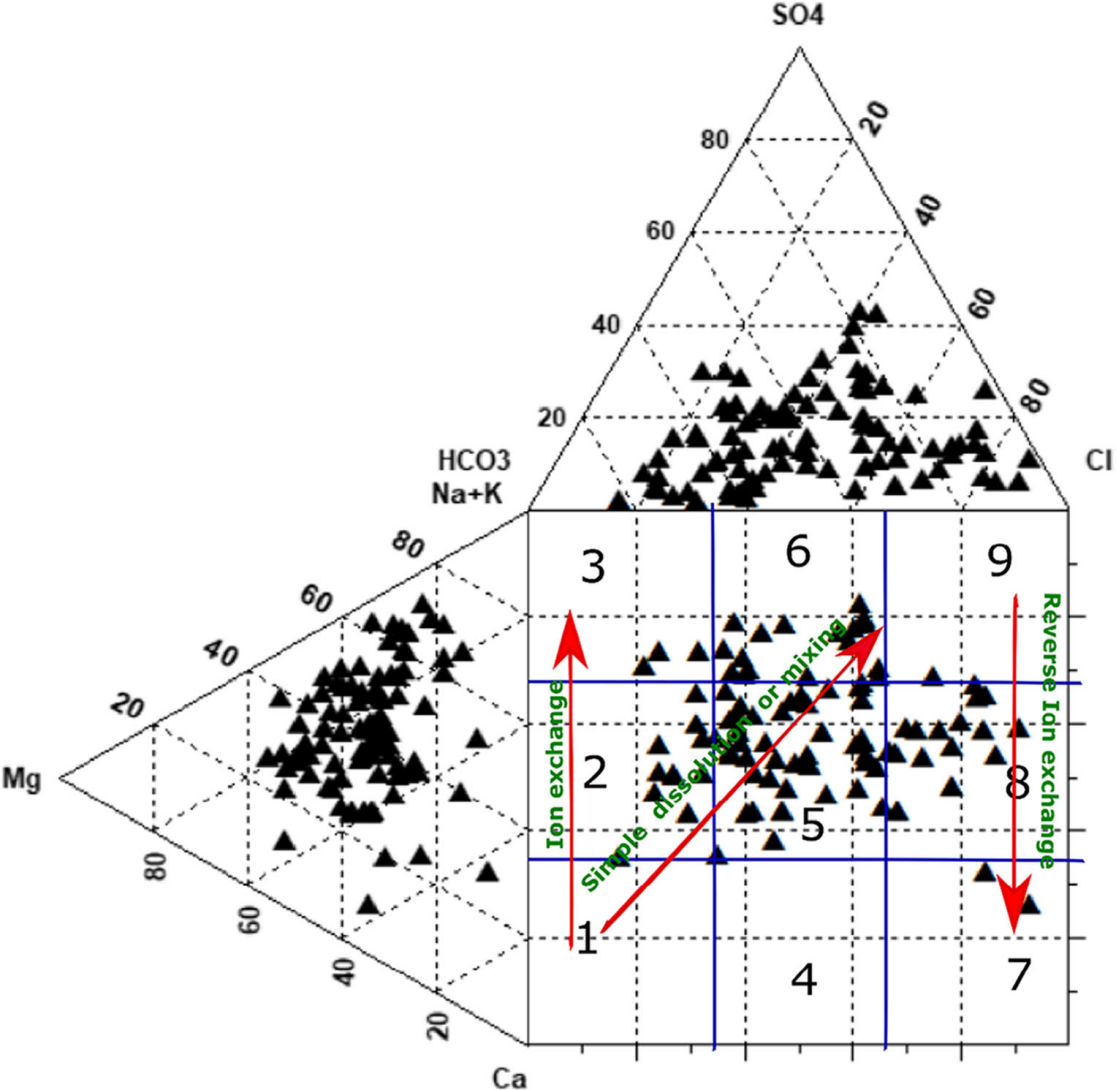
Durov plot showing hydrochemical facies and possible ionic reactions in the study area

A Durov diagram can reflect general geochemical processes in groundwater (Durov, 1948) (Fig. 3). It is also useful in identifying the hydrochemical types of different water samples (Li et al. 2014). In this study, most of the samples were in fields 2, 3, 5, 6, and 8. Exceptionally few samples were plotted in field 7. Field 1 shows the natural groundwater chemistry dominated by Ca and HCO_3_. As it goes from field one to three, significant ion exchange between the cations is expected. Field 2 generally represents the dominance of Ca and Mg along with HCO_3_, while in field 3, Na and K dominate the cations and HCO_3_ the anions. In this study, nine samples fall in field 2, reflecting the dominance of Ca and Mg, while three samples fall in field 3, showing the control of Na and K ions. Many samples were also plotted in field 5, which indicates simple mixing or dissolution processes; in this group, there is no dominance of any anions or cations (Chen et al. 2019). Field 7 shows the dominance of Ca and Cl, generally due to the reverse ion exchange process of Ca from groundwater with Na in the aquifer matrix, while field 8 has a dominance of Ca and Mg along with Cl in several samples, which is indicating the mixing of saline water with the freshwater.

### Nitrate concentration and possible origins in groundwater

The levels of nitrate in groundwater varied between 1 and 415 mg/L, with the average value being 77 mg/L. The permissible limit of nitrate in drinking water, as per WHO guidelines, is 50 mg/L (WHO 2017). Of the 92 samples, 34 exceeded this permissible level for drinking purposes. However, the standard Indian value is lower, at 45 mg/L, making a total 35 samples exceeding this limit. Drinking water standards may vary from place to place depending on water intake related to climatic conditions.

The origin of nitrate is rarely reported from the geological formations. However, the possible origin of nitrate via microbial nitrification can be proposed as the only source of nitrate from natural processes. In this process, ammonia is initially converted to nitrite and then to nitrate with bacterial activity, as shown in Eq. 10 and Eq. 11.

10 $$ {2\mathrm{NH}}_4+{4\mathrm{O}}_2={2\mathrm{NO}}_2+{4\mathrm{H}}_2\mathrm{O} $$

11 $$ {2\mathrm{NO}}_2+{\mathrm{O}}_2={2\mathrm{NO}}_3 $$

An abnormal level of nitrate concentration (> 10 mg/L) must originate from anthropogenic inputs (Panno et al. 2006). The non-point sources, such as agricultural practices, and localized factors, such as sewage leakages, generally control nitrate concentration in surface- or shallow- level groundwater (Wang et al. 2017). It has been observed that higher levels of contamination are mostly associated with shallow groundwater and are the result of cultivation models, manures, wastewater irrigation, redox conditions, precipitation surpluses, waste disposal networks, animal wastes, industry, river–aquifer interactions, denitrification, and related biogeochemical processes in groundwater (Jia et al. 2020; Taufiq et al. 2019; Zendehbad et al. 2019). In the study area, farming activities and farm animals could be the biggest sources of nitrate in groundwater. A large quantity of fertilizer and manures are used in the farming period, usually from February to August, and are leaked into the groundwater with the precipitation surplus. Another interesting source of nitrate was reported by Wakida and Lerner (2005), urbanization and the construction of new buildings which are likely to disturb the soil and soil aeration and develop an atmospheric contract with the nitrogen-fixing microorganisms. The net result if this may increase nitrate mineralization in soil and then increase levels in groundwater.

**Fig. 4:**
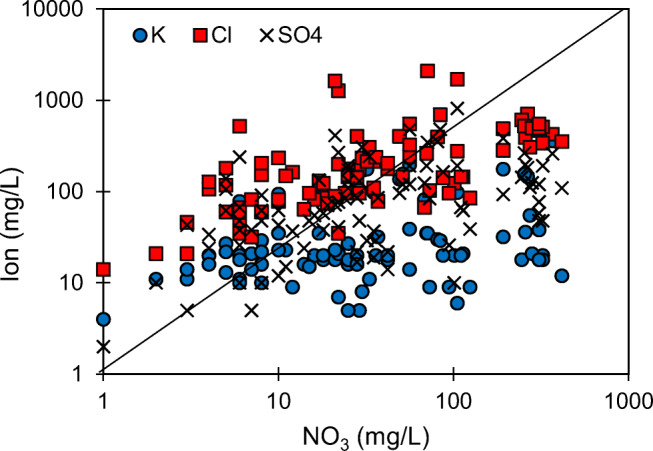
Relationship between NO3 and K, Cl, and SO_4_ (a logarithmic scale is used for better visualization)

Ionic correlation plots were drawn to identify the sources of NO3 in the study area. In this plot, there are many samples that have positively varied for NO_3_ with respect to K, SO_4_, and Cl (Fig. 4). The samples with a positive correlation for NO_3_ with K and SO_4_, indicating the origin of these ions, derived from a non-point source of pollution, including fertilizers (Papazotos et al. 2019). While several samples do not show any positive relation, nitrate is generally controlled by geogenic factors, typically rock–water interaction and weathering of silicate or sulfate minerals. The low concentrations of K in some places can be attributed to the adsorption of K ions by clay minerals (Sajil Kumar et al. 2014).

**Fig. 5:**
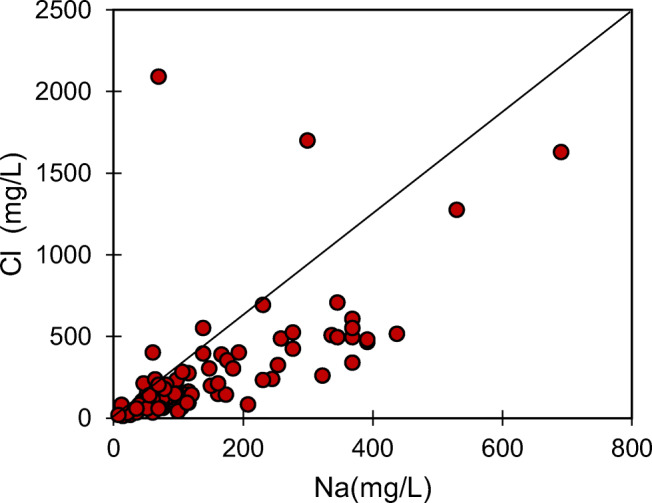
Relationship between Na and Cl in the groundwater

**Fig. 6:**
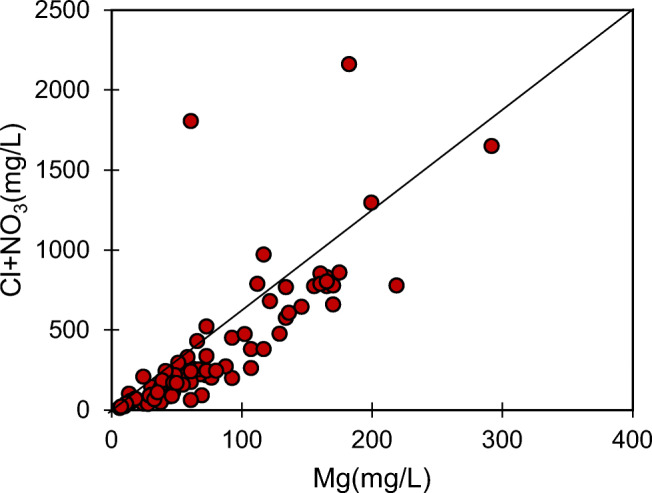
Relationship between Mg and Cl+NO_3_ in the groundwater

Similarly, in the inland regions, the presence of Cl ions in groundwater is an indicator of anthropogenic pollution. There are several samples plotted away from the 1:1 line of NO_3_ vs. Cl, indicating that the origin is not the same for these ions in those samples. The primary source of Cl in the study area is industrial effluents rich in common salt (NaCl), and it is reflected from the substantially high positive concentration for Na and Cl in some of the samples. Furthermore, a very high positive correlation between Na and Cl in Fig. 5 confirms the origin of salinity as highly saline effluent wastewater. However, other sources of Na, like rock weathering, contribute to the total sodium concentration in water, which is evident from some of the samples in which there is no correlation for Na and Cl. These samples represent the natural origin of Na and the possibility of cation exchange with Ca, as observed in the Durov plot. On the other hand, there is a positive correlation of Cl in some of the samples with NO_3_, indicating their common origin from animal and human wastes (Wang et al. 2017). In Fig. 6, the relationship between Mg and Cl+NO_3_ is plotted to understand the origin of NO_3_. An excellent positive correlation between these ions suggests the contribution of agrochemical products (Papazotos et al. 2019). In this plot, it can be observed that the Mg concentrations in some of the samples are higher than the Cl+NO_3_ concentrations, reflecting other sources such as rock weathering.

### Spatial variation of nitrate and land use influences

**Fig. 7:**
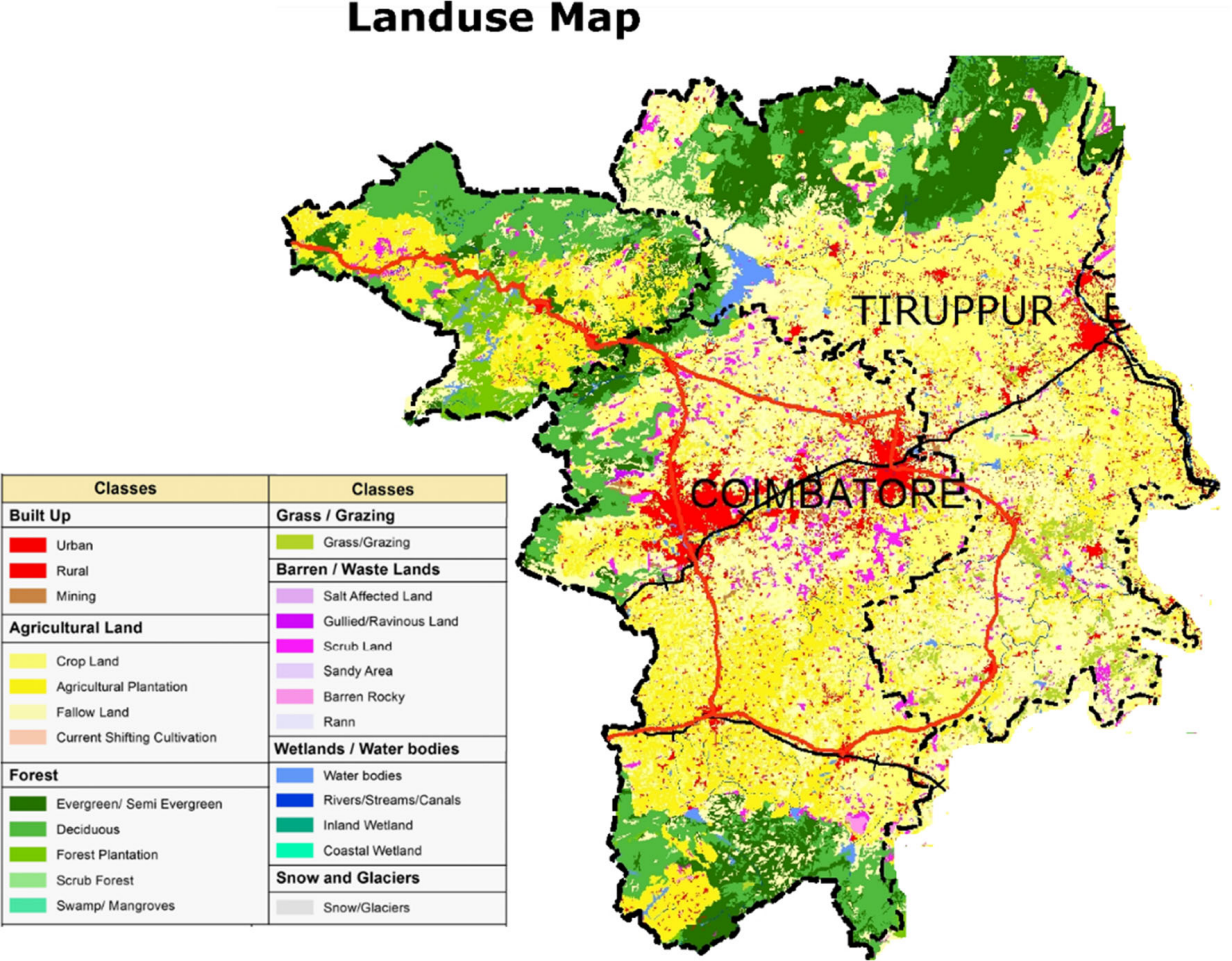
Land use map of the study area

**Fig. 8:**
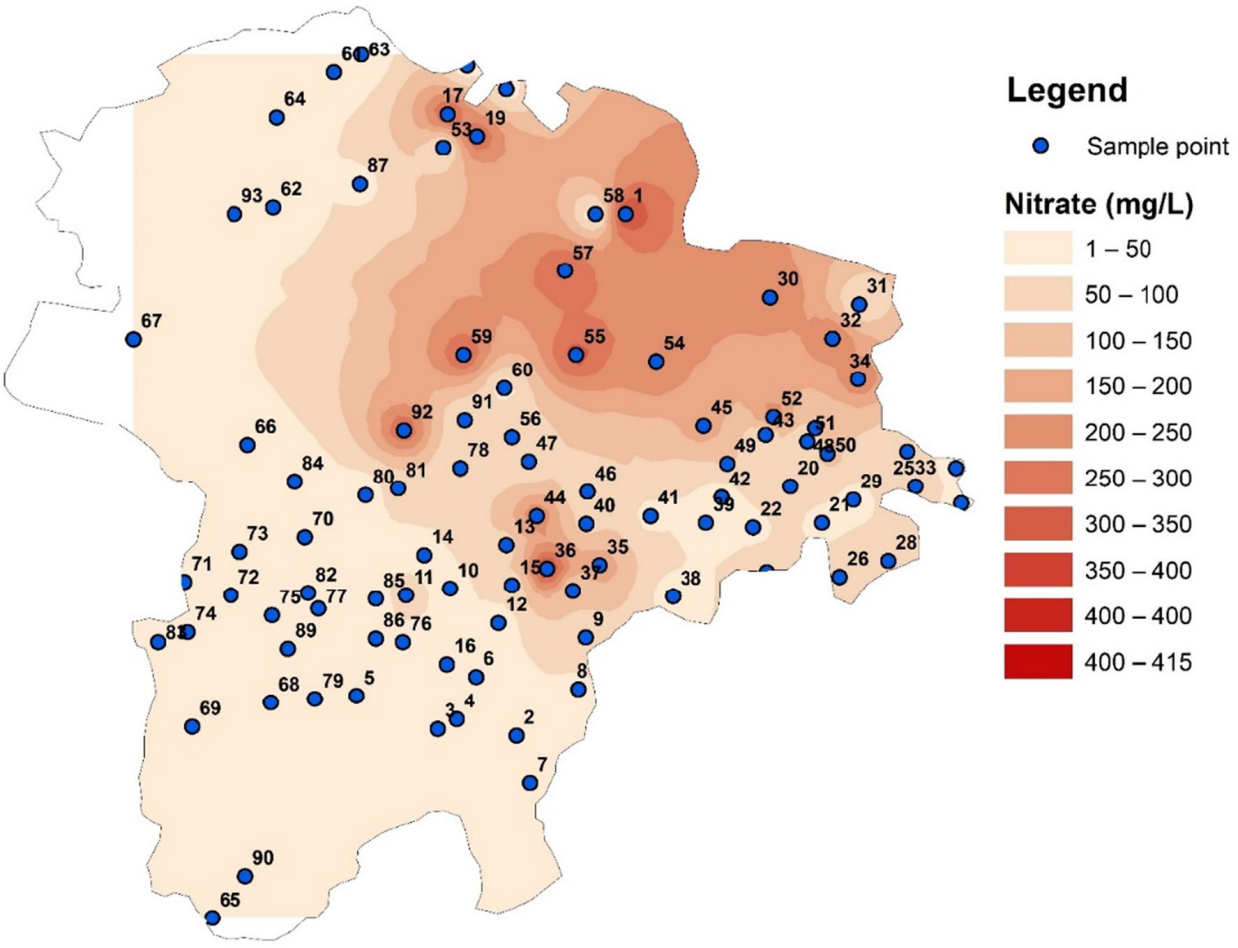
Spatial variation of NO_3_ in the groundwater of the study area

The primary land use patterns are agricultural and industrial with human settlements (Fig. 7). Tirupur is especially famous for textile production, and the exports from this region contribute a large portion of the country’s economy. On the other hand, these industries are polluting the environment, and many regions have been contaminated to an irreversible extent. Variation in nitrate concentration in the study area is shown in Fig. 8, where it can be observed that the concentration is within the permissible limit of 50 mg/L in the majority of the Coimbatore district, while, in contrast, large areas in the Tirupur district have a very high concentration of NO_3_ in groundwater. The spatial dissemination of NO_3_ is largely related to the fertilizer application rate and the efficiency of plants, cropping patterns and cycles, irrigation models, soil structures and textures, and finally, changes in regional climate (Jacks and Sharma 1983; Suthar et al. 2009). The spatial variation maps show that the eastern and northeastern parts of the study area have elevated NO_3_ concentrations.

It has been observed that the hilly regions (recharge areas) have low NO_3_ values; on the other hand, along the flow paths of the Noyyal river, the values are increasing. Nitrate levels in groundwater are mostly controlled by the land use patterns of the region (Lockhart et al. 2013). As shown in Fig. 7, the land use map of the study area shows that large portions of the study area are used primarily for agricultural activities. Forest land and human settlements are the next most prominent land uses. In agricultural areas, it can be observed that the application of fertilizer is higher than the required level. In this study area, the surface irrigation is generally practiced, which results in uneven distribution of water and sometimes results in irrigation return flows. Growing crops like paddy, maize, vegetables, and grain needs seasonal tillage, which alters the soil structure and its ability to activate microbial fixation of nitrate, especially in the bean-cultivating regions. The human settlements in the eastern part of the study area are the result of the fertile soil for agriculture (SPA 2008). In addition, the exponential growth of settlements generates a substantial amount of human and animal waste, also causing nitrate contamination in the groundwater of the study area. In several locations, sanitation facilities are inadequate or unavailable. It is causing the leaching of nitrate into soil, which is partially consumed by plants and finally reaching the groundwater.

### Human health risk assessment of nitrate

Health risk assessment is the quantification of harmful health effects in humans due to continuous excess exposure to toxic chemicals in the environment (Şener et al. 2017). Table 4 shows a summary of the statistics for the health risk assessment of nitrate for males, females, and children. The chronic daily intake (CDI) values of nitrate for males varied between 0.04 and 15.96 mg/kg/day, with an average of 2.96 mg/kg/day; CDI for females varied between 0.05 and 18.87 mg/kg/day, with an average of 3.5 mg/kg/day, and for children, it was between 0.05 and 21.58 mg/kg/day with an average of 4.00 mg/kg/day. CDI values were further used in determining the hazard quotient via ingestion.

**Table 4:** Results of health risks of nitrate contamination via dermal and ingestion in children and adults

S. No	Chronic daily intake (CDI)			Dermally absorbed dose (DAD)			HQ_Oral_			HQ_Dermal_			Hazard index (HI)		
	Male	Female	Child	Male	Female	Child	Male	Female	Child	Male	Female	Child	Male	Female	Child
Min.	0.04	0.05	0.05	0.00	0.00	0.00	0.02	0.03	0.03	0.00	0.00	0.00	0.02	0.03	0.03
Max.	15.96	18.87	21.58	0.04	0.05	0.13	9.98	11.79	13.49	0.03	0.03	0.08	10.00	11.82	13.57
Avg.	2.96	3.50	4.00	0.01	0.01	0.02	1.85	2.19	2.50	0.00	0.01	0.02	1.85	2.19	2.52
% exceeded							40	42	45	0	0	0	40	42	45

The dermally absorbed dose (DAD) was calculated as a step of determining the HQ_Dermal_. DAD values of nitrate for males varied between 0.0 and 0.04 mg/kg/day, with an average of 0.01 mg/kg/day. DAD for females varied between 0.0 and 0.05 mg/kg/day, with an average of 0.01 mg/kg/day. For children, DAD ranged between 0.00 and 0.13 mg/kg/day with an average of 0.02 mg/kg/day.

In this study, HQ through the oral pathway for men varied between 0.024 and 9.9. The average value is 1.85, which is higher than the recommended value of 1; thus, a non-carcinogenic risk is visible for men in the study area. Sample 36 from the Munduelanpatty village showed the highest value at 9.9, followed by sample 1 in the Timmanaickanpalayam village which was measured at 8.79. From a total of 37 samples, 40% exceeded the permissible value. Slightly higher values, between 0.03 and 11.79, were observed in HQ_Oral_ for females, with an average value of 2.19. Thirty-nine samples (42%) had an HQ_Oral_ higher than 1 for females. Among the three categories studied, children have the highest values of HQ_Oral_, varying between 0.03 and 13.57, with an average of 2.52. In total, 44 samples (45%) exceeded the limit and pose severe health impacts for children. In this study, HQ_Dermal_ was less than 1 in all the samples, and thus does not considerably impact human health.

**Fig. 9:**
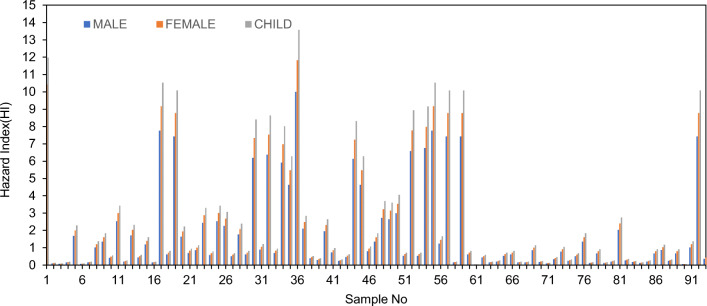
Hazard index (HI) of nitrate in individual samples for male, female, and children

As shown in Table 3, the cumulative HI of males ranges between 0.024 and 10.00, with an average of 1.90. In the case of females and children, the range is from 0.028 to 11.825 and 0.032 to 13.57 respectively, with a mean value of 2.24 for females and 2.57 for children. In the evaluation, the average cumulative hazard index for males, females, and children is higher than the recommended value of 1, thus revealing the high non-carcinogenic risk in the study area due to nitrate concentration. The value of HI for children is higher than for females or males and is in the order HI_child_˃HI_female_˃HI_male_ (Fig. 9). Therefore, it is clear that children are more vulnerable to non-carcinogenic risks due to nitrate toxicity. The basic reason for this is that their water consumption is more per unit of their body weight than adults (Chen et al. 2016; Li et al. 2019). The HI for children was found to be the highest in sample 36 (Munduvelampaty village), followed by sample 1 (Timmanckanpalayam village). Villages Tundarkarampalayam, Sevur, Kanjeerapalayam, Vellakoil, MuthunaickkenValasu, Periakumarapalayam, Uthgiyur, Kambliyampatti, AvinashipalayamSouth, Pongalur, Veerapadi, K.Ayyampalayam, and Vadavalli also showed very high values of HI for children.

**Fig. 10:**
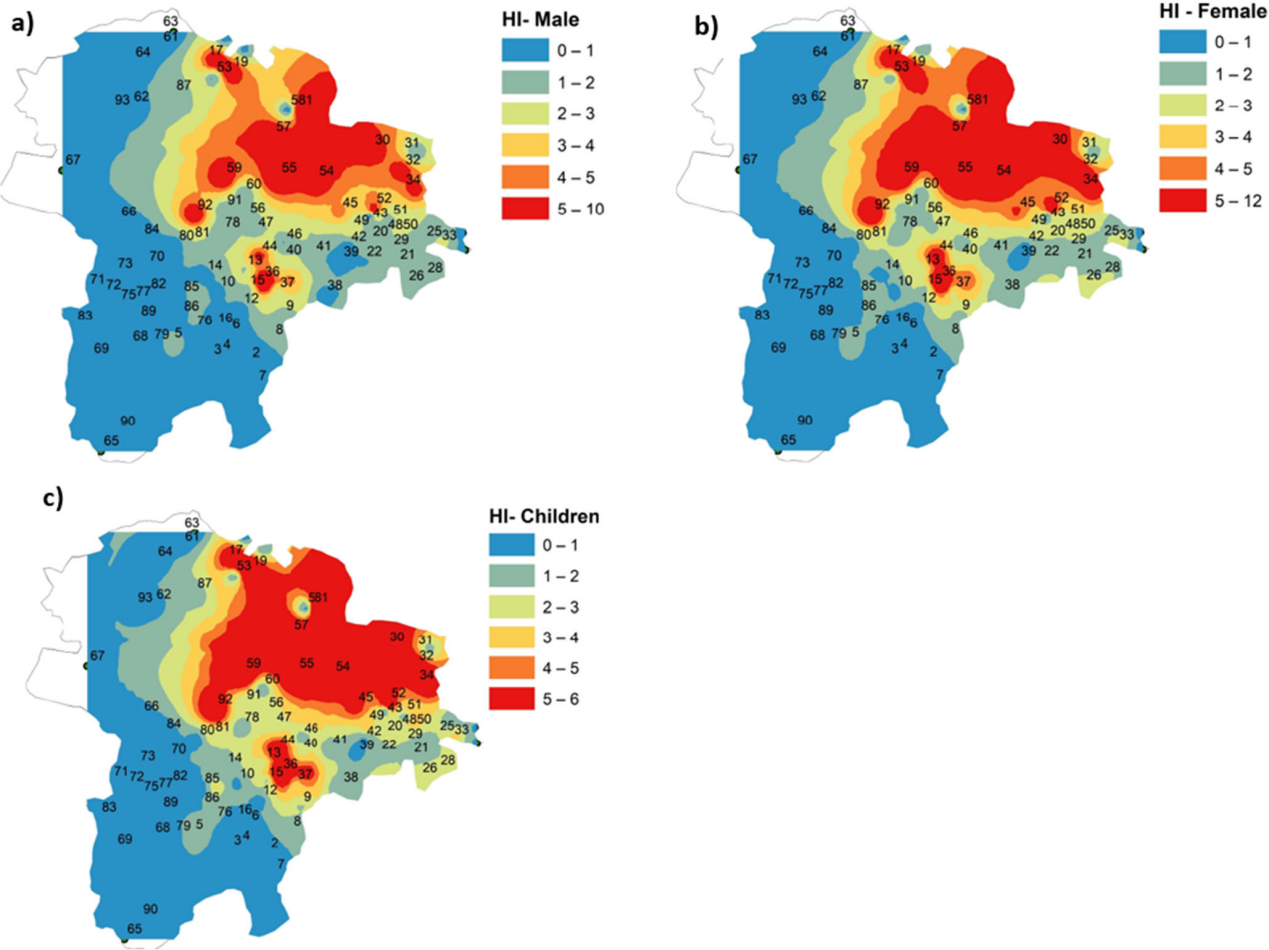
Spatial variation of hazard index (HI). **a** Males. **b** Females. **c** Children

The spatial variation of HI values was plotted for males, females, and children in Fig. 10a, b, and c. It can be observed from the plot that the groundwater quality is heavily polluted in the north and northeastern parts of the study area. As explained in the earlier section, this is mainly due to intensive agricultural practices. The spatial maps show that the percentage of critical regions was predicted to be higher than that observed in the statistical summary. From males to children, the values of HI were found to increase from the central part of the study area to the northeastern part, though the health risk can be expected to spread to the southern part of the region as the land use pattern of the study area changes due to government promotion of more intensive agricultural practices. This will mean an accelerated use of fertilizers and manures, which will then increase the NO_3_ concentration in the groundwater. In this situation, there is an urgent call for tackling the problem of ensuring a supply of safe drinking water for all the villages, a need which requires proper monitoring of water quality in contaminated sites.

## Conclusions

Groundwater quality as related to nitrate contamination has been studied in the Coimbatore and Tirupur districts using classical methods such as bivariate plots and Durov diagrams. Based on the hydrochemistry of the groundwater samples, the results of this study support the existence of natural and anthropogenic influences on water quality. Nitrate levels in the study area varied between 1 and 415 mg/L, with an average value of 77 mg/L. In total, 37% of the samples exceeded the WHO drinking water limit of 50 mg/L. It is observed that the origin of nitrate can be primarily attributed to the overuse of agricultural fertilizers and to leaks in urban sewage and septic tanks resulting from exponential population growth. Risk from exposure to nitrate (HQ_Dermal_ < 1 in all the samples) has not been found in the study area; however, there is a possible health risk due to ingestion. The HQ via ingestion exceeded one in 40% of males, 42% of females, and 45% of children. The HI was the same as the HQ_oral_, as there are no health issues related to the dermal pathway. The order of human health risk is children˃females˃males; even with the same concentration of nitrate in drinking water, children are more prone to illness because body weight has been found to be the determining factor causing variation in health risk in these three categories. According to the spatial variation of HI values, the highest health risk exists in the north and northeastern parts of the study area and can be attributed to intensive agricultural practices.

## Data Availability

The datasets used and/or analyzed during the current study is available from the corresponding author on reasonable request.
