# Correction to: Exposure and health risk assessment of nitrate contamination in groundwater in Coimbatore and Tirupur districts in Tamil Nadu, South India

**DOI:** 10.1007/s11356-021-17079-0

**Published:** 2021-10-20

**Authors:** Sajil Kumar Pazhuparambil Jayarajan, Lemoon Kuriachan

**Affiliations:** 1 Institute of Geological Sciences, Hydrogeology Group, Freie Universität Berlin, Malteserstr. 74-100, 12249 Berlin, Germany; 2 Water Institute, Karunya University, Coimbatore, 641114 India

**Correction to: Environmental Science and Pollution Research (2021) 28:10248–10261**

**https://doi.org/10.1007/s11356-020-11552-y**

The article Exposure and health risk assessment of nitrate contamination in groundwater in Coimbatore and Tirupur districts in Tamil Nadu, South India by Sajil Kumar Pazhuparambil Jayarajan and Lemoon Kuriachan, was originally published online on 10 November 2020 without open access. With the author(s)’ decision to opt for Open Choice the copyright of the article changed on 12 October 2021 to © The Author(s) 2021 and the article is forthwith distributed under a Creative Commons Attribution 4.0 International License, which permits use, sharing, adaptation, distribution and reproduction in any medium or format, as long as you give appropriate credit to the original author(s) and the source, provide a link to the Creative Commons licence, and indicate if changes were made. The images or other third party material in this article are included in the article’s Creative Commons licence, unless indicated otherwise in a credit line to the material. If material is not included in the article’s Creative Commons licence and your intended use is not permitted by statutory regulation or exceeds the permitted use, you will need to obtain permission directly from the copyright holder. To view a copy of this licence, visit http://creativecommons.org/licenses/by/4.0.

The Original article has been corrected.

